# A case report of primary hepatoid adenocarcinoma of the vagina and literature review

**DOI:** 10.3389/fonc.2026.1799019

**Published:** 2026-04-20

**Authors:** Mingyuan Yuan, Qige Xu, Zhaoyuan Niu, Chengxiang Gao

**Affiliations:** 1 Department of Gynecology, Jiaozhou Central Hospital of Qingdao, Qingdao, China; 2 Department of Pathology, Jiaozhou Central Hospital of Qingdao, Qingdao, China; 3 Department of Gynecology, The Affiliated Hospital of Qingdao University, Qingdao, China

**Keywords:** alpha-fetoprotein, case, hepatoid adenocarcinoma, primary, vagina

## Abstract

Hepatoid adenocarcinoma (HAC) is a rare extrahepatic malignant tumor that exhibits histological features resembling those of hepatocellular carcinoma. It is typically accompanied by an abnormal elevation in serum alpha - fetoprotein (AFP) levels. HAC can occur in multiple organs, with the stomach being the most common site. It has also been reported in organs such as the esophagus, colon, pancreas, lung, ovary, and uterus. After reviewing the relevant literature, to date, there have been no reports of primary HAC in the vagina. This article presents the first case of primary vaginal hepatoid adenocarcinoma. The patient was a post - menopausal elderly woman. By presenting the characteristics of the disease onset, clinical manifestations, clinicopathological features, molecular characteristics, and the treatment process, and through a literature review, the objective is to improve the understanding of this rare disease and offer a reference for clinical diagnosis and treatment.

## Case presentation

A 75-year-old female patient presented to the clinic on February 22, 2022, with a chief complaint of vaginal bleeding lasting for one week. Her past medical history included a left mastectomy for breast cancer 20 years prior, followed by intravenous chemotherapy. Ten years ago, she underwent a laparoscopic hysterectomy and bilateral salpingo-oophorectomy at another hospital for a “benign uterine tumor” (details unspecified). Menarche occurred at age 18 with regular cycles, and menopause at age 49. There was no significant family history of malignancy or related diseases.

**Figure 1:**
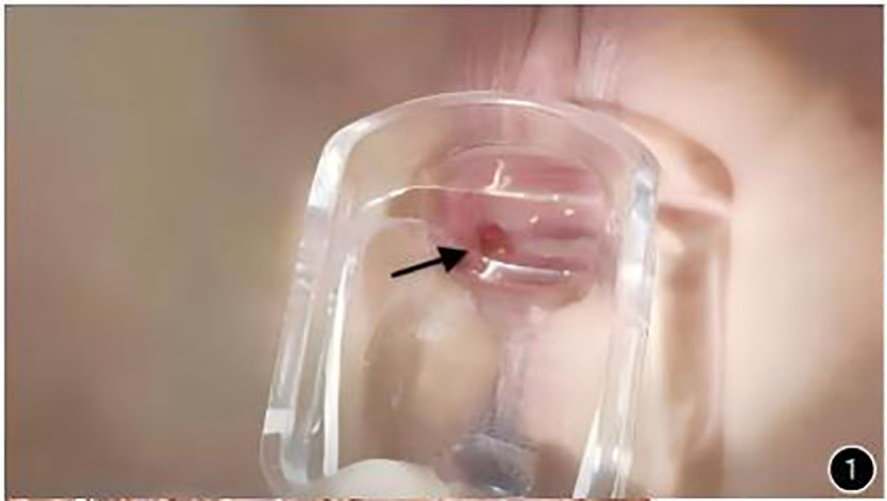
Gynecological examination findings: cervix absent. Vaginal vault well healed. A 1cm * 0.8cm polypoid mass observed on the right wall of the mid-vagina, smooth surface, fragile texture.

Gynecological examination revealed vulvar atrophy. A 1.0 cm × 0.8 cm bright red, friable, exophytic lesion with contact bleeding (+) was observed on the mid-right lateral vaginal wall (**Figure 1**). The cervix was absent, the vaginal cuff was well-healed, and the pelvis was empty without palpable masses. Rectal examination was unremarkable. TCT and HPV co-testing showed no abnormalities. The vaginal lesion was excised and sent for pathological examination.

**Figure 2:**
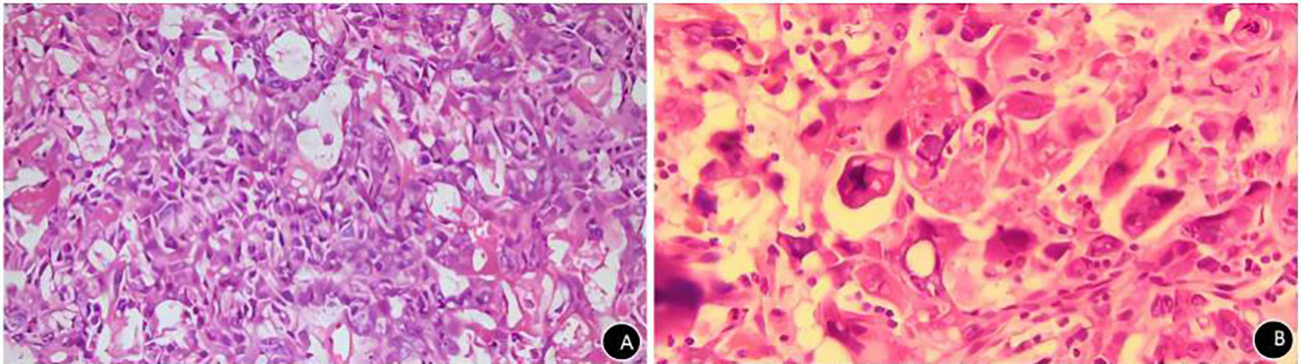
Histopathological examination of vaginal hepatoid adenocarcinoma specimen. **(A)** Tumor cells arranged in adenoid and solid patterns, showing significant atypia. Nuclei are vesicular, most with prominent nucleoli. Cytoplasm is abundant and eosinophilic. Tumor exhibits rich internal sinusoids, displaying features of hepatocellular and adenocarcinomatous differentiation. HE×200. **(B)** Tumor giant cells and mitotic figures readily seen. HE×400.

**Figure 3:**
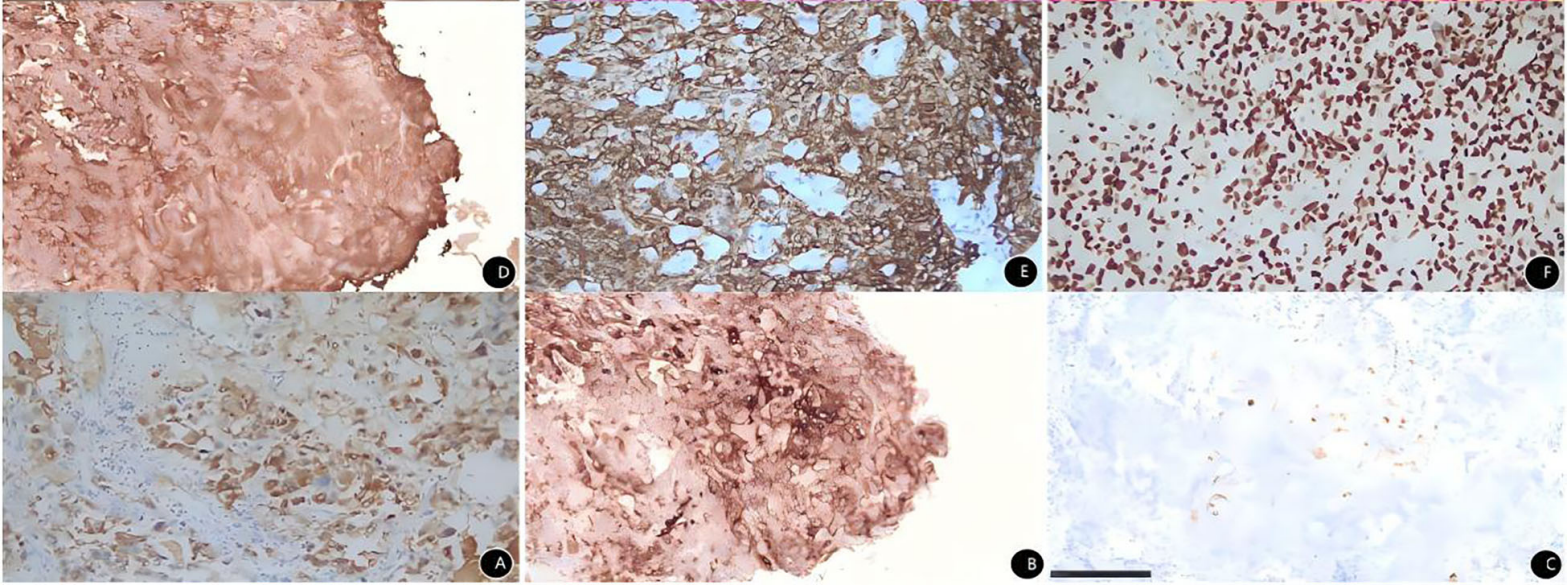
Immunohistochemical results of vaginal hepatoid adenocarcinoma specimen. **(A)** Positive AFP expression in tumor cells. EnVision method, medium magnification. **(B)** Positive Glypican-3 expression in tumor cells. EnVision method, medium magnification. **(C)** Positive HepPar-1 expression in tumor cells. EnVision method, medium magnification. **(D)** Positive α1-AT expression in tumor cells. EnVision method, medium magnification. **(E)** Positive CK expression in tumor cells. EnVision method, medium magnification. **(F)** Positive SALL4 expression in tumor cells. EnVision method, medium magnification.

Histopathological analysis revealed a malignant tumor with glandular and solid growth patterns. The tumor cells exhibited significant atypia, abundant eosinophilic cytoplasm, frequent giant cells and mitotic figures, and prominent intratumoral blood sinuses. Immunohistochemistry (IHC) supported the diagnosis of poorly differentiated hepatoid adenocarcinoma (**Figure 2**). IHC results were as follows (**Figure 3**): The immunohistochemical markers related to hepatocellular carcinoma (AFP, glypican-3, HepPar-1, α1-AT) were all positive. Additionally, SALL4, CK and ERG were positive, and Ki-67 was highly expressed. CEA, GATA3, P16, CA125, SOX10, GCDFP-15, AR, PLAP and CD10 were all negative.

One week post-excision, the vaginal bleeding ceased.

On March 1, A PET-CT scan showed no evidence of residual or metastatic disease. Surgical intervention was recommended but declined by the patient.

On May 7, 2022, the patient returned with “vaginal bleeding for 3 days.” Examination revealed a recurrent friable, bleeding lesion (0.8 cm × 0.5 cm) at the same vaginal site.

On May 9, 2022. Laboratory findings: Serum AFP 203.39 ng/mL (normal: 0–7.02), CEA 6.27 ng/mL (0–3.4), IL-5 6.33 pg/mL (0–3.1), IL-6 7.24 pg/mL (0–5.4), IL-1β 23.69 pg/mL (0–12.4), IL-8 432.43 pg/mL (0–20.6). Other tumor markers (CA125, HE4, CA19-9, β-HCG, SCC-Ag) and interleukins were within normal limits.

Admission diagnoses were: (1) Vaginal malignant tumor (hepatoid adenocarcinoma), Stage I; (2) Postoperative status for breast cancer; (3) Status post total hysterectomy.

After completing preoperative evaluations and excluding surgical contraindications, the patient underwent wide local excision of the vaginal lesion under general anesthesia on May 10, 2022. The resection margin was 1 cm from the lesion edge, with a depth of 0.5 cm. Postoperative pathology confirmed poorly differentiated hepatoid adenocarcinoma with clear margins.

Genetic analysis of the tumor tissue was performed using target-capture next-generation sequencing, revealing the following alterations: TP53 (c.742C>T, p.R248W), NF1 splice-site variant (c.6705-6711del), MDM4 copy number amplification, FGFR3 copy number amplification, and an AR substitution mutation (c.1199C>T, p.A400V).

Postoperative recovery was uneventful. One week after surgery, serum AFP decreased to 42.19 ng/mL.

Adjuvant radiotherapy was administered as follows: From July 1 to August 4, 2022, regional pelvic and inguinal lymph nodes received 50 Gy in 25 fractions using 6-MV X-rays. From August 5 to August 12, 2022, an additional vaginal cuff boost of 10 Gy in 2 fractions was delivered to a 3 cm length and 5 mm submucosal depth.

Serum AFP normalized one month after completing radiotherapy. The patient has been followed for 36 months with no evidence of tumor recurrence and persistently normal serum AFP levels (**Table 1**).

**Table 1:** Clinical timeline of treatment and follow-up.

Date / period	Event / milestone	AFP level (ng/ml)	Notes
Single time points			
2022-02-22	Initial surgery	–	–
2022-05-07	Recurrence	203.39	–
2022-05-10	Reoperation	–	–
2022-09-13	Follow-up; no recurrence observed	5	AFP returned to normal level.
Time periods			
2022-07–01 to 2022-08-04	Pelvic Radiotherapy (Inclusive of Inguinal Region)	–	Post-reoperation adjuvant therapy.
2022-08–05 to 2022-08-12	Intracavitary Vaginal Brachytherapy	–	Post-reoperation adjuvant therapy.
2022-12–01 to 2025-10-01	Follow-up; no recurrence observed	0-5	Long-term surveillance with normal AFP range.

AFP, Alpha-fetoprotein.

## Discussion

Hepatoid adenocarcinoma (HAC) is a rare, special type of adenocarcinoma, defined as a tumor with hepatocellular differentiation occurring in extrahepatic organs or tissues. Its incidence is 0.014 per 100,000 (1). First reported in the gastrointestinal tract in 1970, it was also termed AFP-producing gastric adenocarcinoma (2). In 1985, Ishikura first proposed the concept of hepatoid adenocarcinoma (3). To date, HAC has been reported in multiple organs, with the gastrointestinal tract and other digestive organs being the most common sites. Occurrence in the female reproductive system is particularly rare. Literature review up to the submission date in 2025 indicates over 40 cases primary to the ovary (4), 20 primary to the endometrium (5), 4 primary to the fallopian tube (6), and only 3 primary to the cervix. There have been no reports of primary vaginal HAC, with only one case reported in the urethra and vaginal septum, adjacent to the vagina (7). Primary vaginal cancer typically occurs in elderly postmenopausal women, with main clinical manifestations being irregular vaginal bleeding and discharge. It is closely associated with persistent infection with high-risk HPV, especially type 16 (8). The predominant histological type is squamous cell carcinoma; other types of vaginal cancer are uncommon. This case of hepatoid adenocarcinoma represents the first reported pathological type of primary vaginal cancer. Its age of onset and clinical symptoms are non-specific. Routine HPV testing suggests the etiology of this pathological type is not related to HPV infection. The pathogenesis remains unclear, and the tissue origin is complex, possibly involving abnormal differentiation of pluripotent stem cells (9), dedifferentiation or transdifferentiation (10), abnormal embryonic development (11–13), and regulation of molecular signaling pathways (14). Although some studies support these hypotheses, further exploration of the specific mechanisms is needed.

Symptoms of HAC are non-specific and depend on the tumor location. There are no specific imaging characteristics. However, elevated serum alpha-fetoprotein (AFP) is more common than in conventional adenocarcinomas, which may aid in early diagnosis. Additionally, AFP is an independent prognostic factor for HAC. One study indicated that in HAC, preoperative serum AFP levels ≥500 ng/ml were closely associated with poorer overall survival (OS) (15). Interleukins, as immune modulators, participate in the regulation of the tumor microenvironment. Certain interleukins (such as IL-6, IL-8, IL-10) are significantly elevated in tumor patients and can serve as auxiliary diagnostic markers. In this case, interleukin-5 (IL-5), interleukin-6, and interleukin-1β (IL-1β) were mildly elevated, while interleukin-8 (IL-8) was markedly elevated. Current research has confirmed that IL-8 is closely related to the diagnosis, treatment, and prognosis of various malignant tumors including liver cancer (16), ovarian cancer (17), breast cancer (18), lung cancer (19), colon cancer (20), and cervical cancer (21), and can assist in diagnosing malignancies. Due to the lack of studies related to vaginal cancer, its significance in vaginal cancer remains unclear.

Histopathology is currently the gold standard for diagnosing HAC. Typically, HAC histologically exhibits features of both hepatocellular and adenocarcinomatous differentiation. In some cases, hepatoid and adenocarcinomatous areas coexist, forming a unique “biphasic” pattern (22). Tubular and/or papillary adenocarcinoma can be observed in the adenocarcinomatous differentiation areas. Hepatoid differentiation can be seen in tumor tissue arranged in nests, trabeculae, and glandular patterns. Tumor cells have large, polygonal, strongly eosinophilic cytoplasm. Eosinophilic vesicles may be seen in the cytoplasm or stroma, resembling hepatocellular carcinoma. The immunohistochemical profile of HAC is crucial for its diagnosis and differential diagnosis. Alpha-fetoprotein (AFP) is one of the most characteristic markers for HAC, expressed in 91.6% of HAC cases (23), reflecting its hepatocellular differentiation. AFP expression levels may vary among cases, but its positive expression holds significant value for diagnosing HAC. Besides AFP, HAC often expresses other hepatocellular carcinoma-related markers, such as: Glypican-3 (100%), CEA (78.7%), CD10 (62.5%), HepPar-1 (38.1%) (23). SALL4 is an important marker for germ cell tumors. Some studies report that it is usually not expressed in hepatocellular carcinoma but is expressed in HAC (24–26). Cytokeratin, a marker for epithelial tumors, is also commonly expressed. The expression patterns of different CK subtypes help identify the primary site and subtype of the tumor. Its cytokeratin profile resembles that of the related ordinary epithelial adenocarcinoma (related ordinary epithelial immunophenotype CK18+/CK19+/CK20±) (27). The histopathology in this case exhibited features of HAC (**Figure 2**) and presented a typical hepatocellular immunophenotype, with positivity for AFP, glypican-3, HepPar-1, and α1-AT (**Figure 3**). The SALL4 positivity aligns with literature reports of HAC at other sites. Cytokeratin was positive. Unfortunately, CK subtype testing was not performed. The negativity for CEA, GATA3, CA125, and PLAP on IHC argues against tumors of ordinary epithelial or germ cell origin.

Clinically, it needs to be differentiated from yolk sac tumor, metastatic hepatocellular carcinoma, vaginal adenocarcinoma, clear cell carcinoma, etc. 1. Yolk Sac Tumor: A highly malignant germ cell tumor, common in young women, especially children and adolescents. Its morphological features differ from HAC, often showing Schiller-Duval bodies and a reticular pattern. Immunohistochemistry: AFP, Glypican3, SALL4 often positive. 2. Metastatic Hepatocellular Carcinoma: Imaging shows primary lesion in liver parenchyma. Morphological features are very similar to HAC. Positive for AFP, HepPar-1, Arg-1, and Glypican3, but negative for CK7 and CK19. 3. Clear Cell Carcinoma: Occasionally seen in the vagina. Tumor cells have clear cytoplasm, often showing hobnail cells and tubular structures. Usually does not express AFP, HepPar-1, Arg-1, and Glypican3, but may express CA-125 and CK7. 4. Primary Vaginal Adenocarcinoma: Morphological features differ from HAC, often showing glandular structures and mucin production. Immunohistochemistry: Adenocarcinomas typically do not express hepatocellular markers but may express CEA, CA-125, and CK7 (**Table 2**).

**Table 2:** Differential diagnosis of hepatoid adenocarcinoma (HAC) of the vagina.

Feature	Hepatoid adenocarcinoma (HAC)	Yolk sac tumor (YST)	Metastatic hepatocellular carcinoma (HCC)	Clear cell carcinoma (CCC)	Primary vaginal adenocarcinoma (Conventional)
Clinical Context	Rare primary vaginal tumor	Young women, **children and adolescents**	**Known primary liver mass** on imaging	Can occur primarily in the vagina	Primary vaginal adenocarcinoma
Morphology	Hepatoid differentiation (eosinophilic cytoplasm, nested/trabecular growth)	**Schiller-Duval bodies, reticular/microcystic pattern**	Very similar to HAC (hepatoid features)	**Hobnail cells**, tubular-cystic structures	**Glandular structures, mucin production**
AFP	Often +	+	+	–	–
Glypican 3	+	+	+	–	–
HepPar-1	+	– (Usually)	+	–	–
Arg-1	+	– (Usually)	+	–	–
CK7	Often +	– / Variable	– (Key differentiator)	+	+
CK19	Often +	Variable	–	Variable	+
CA-125	–	–	–	+	+
SALL4	Variable	+	–	–	–
CEA	Variable	Variable	– (Usually)	Variable	+

Children and adolescents — Yolk sac tumor (YST) is most commonly seen in this age group, which is a key clinical feature. Known primary liver mass — Indicates metastatic hepatocellular carcinoma (HCC), a critical distinction from primary vaginal hepatoid adenocarcinoma (HAC). Reticular/microcystic pattern — A typical histologic pattern of yolk sac tumor (YST). Schiller-Duval bodies — A characteristic histologic feature of yolk sac tumor (YST), resembling glomeruli and considered pathognomonic. Hobnail cells — A defining cytologic feature of clear cell carcinoma (CCC), characterized by cells with bulbous nuclei protruding into the lumen. Glandular structures, mucin production — Typical histologic features of conventional primary vaginal adenocarcinoma.

Currently, most research on HAC focuses on its clinicopathological features, with fewer studies on its molecular characteristics. With the development and application of next-generation sequencing (NGS) technology, related research has begun to gradually focus on its molecular features. This study used target region probe capture technology and NGS, revealing: TP53 substitution mutation (c.742C>T, p.R248W), NF1 alternative splicing (c.6705-6711del), MDM4 copy number amplification, FGFR3 copy number amplification, AR substitution mutation (c.1199C>T, p.A400V). TP53 (a tumor suppressor gene) is the most common and the only gene mutated across HACs of different sites (14, 28–30). The mutation frequency of TP53 is comparable to that in common gastric adenocarcinoma, with most mutation sites located in the p53 DNA-binding domain (29). Additionally, other gene mutations have been reported in HAC, among which CEBPA, RPTOR, WISP3, MARK1, and CD3EAP are confirmed to have higher mutation frequencies in HAC. The frequency of gene mutations in HAC differs from that in ordinary adenocarcinomas. This study showing TP53 mutation is consistent with research on HAC from different sites. As there is currently no related research on primary vaginal HAC, the MDM4 and FGFR3 gene amplifications in this case are considered potentially related to tumor aggressiveness and poor prognosis. The significance of NF1 and AR remains unclear and requires further exploration.

Due to the rarity of HAC, there is currently no standard treatment regimen. Comprehensive treatment generally involves radical surgical resection combined with adjuvant radiotherapy and/or chemotherapy. Some studies suggest HAC has poor chemotherapy sensitivity (28). Genetic testing can be performed to assess suitability for targeted and immunotherapy. Studies have shown that anti-angiogenic drugs (ramucirumab, trastuzumab) help improve the prognosis of some patients with gastric HAC (31). Some scholars have reported that sorafenib can provide short-term clinical benefit in treating advanced recurrent HAC (32). This patient exhibited high serum IL-8 expression, which is associated with angiogenesis, metastasis, and drug resistance, representing a potential therapeutic target. It also provides a rationale for exploring targeted therapies against IL-8 and its signaling pathway for HAC, such as IL-8 monoclonal antibodies (e.g., BMS-986253), CXCR1/CXCR2 inhibitors (e.g., Reparixin, Navarixin, SX-682), and downstream signaling pathway inhibitors (e.g., PI3K inhibitors, NF-κB inhibitors). This requires further research for confirmation. Primary vaginal hepatoid adenocarcinoma has not been previously reported. The treatment for this case was referenced against the NCCN Clinical Practice Guidelines for vaginal cancer. After surgical resection of the vaginal lesion, and considering the patient’s age, tumor stage, and treatment preferences, postoperative radiotherapy combining intracavitary and external beam irradiation was administered, without chemotherapy. Literature review revealed that Fan Y et al. reported a case of hepatoid adenocarcinoma in the urethrovaginal septum in May 2023, with a primary site close to this case. After lesion resection, the patient received adjuvant concurrent chemoradiotherapy, including three cycles of gemcitabine, cisplatin, and bevacizumab along with concurrent pelvic radiotherapy (25 fractions). The patient achieved disease-free survival for 6 months (7). Due to the rarity of such cases, clinical experience is relatively limited, and further in-depth research is needed to accumulate more effective data. HAC generally exhibits strong invasiveness, early metastasis tendency (e.g., to liver, lung), and lacks effective treatment modalities, leading to an overall poor prognosis (31, 33). Long-term close follow-up is essential. This patient experienced recurrence only 3 months after initial lesion excision. Prompt surgery and adjuvant radiotherapy were performed subsequently. The patient has now achieved disease-free survival for over 36 months and continues under close follow-up. Therefore, early diagnosis and comprehensive treatment remain key to improving prognosis.

## Conclusion

In summary, primary vaginal hepatoid adenocarcinoma is extremely rare clinically, and this case represents the first report. It occurred in an elderly postmenopausal woman, with initial symptoms of irregular vaginal bleeding. Preoperative diagnosis is challenging, relying mainly on histopathological confirmation, with immunohistochemistry assisting in differential diagnosis. Surgical resection is the primary treatment method. Postoperative radiotherapy and/or chemotherapy can be administered based on tumor stage, differentiation degree, AFP levels, etc. Genetic testing results provide possibilities for subsequent targeted and immunotherapy. Due to the rarity of such cases, continued accumulation of diagnosis and treatment experience is necessary for further research.

## Data Availability

The raw data supporting the conclusions of this article will be made available by the authors, without undue reservation.
